# A Case Report and Literature Review of Skeletal Muscle Metastasis of Non-small Cell Lung Cancer

**DOI:** 10.7759/cureus.54967

**Published:** 2024-02-26

**Authors:** Linda Youn, Mirra Srinivasan, Amber Kuta, Jacyln Barnard, Mazen Khalil

**Affiliations:** 1 Internal Medicine, St. Bernards Medical Center, Jonesboro, USA; 2 Internal Medicine, California Institute of Behavioral Neurosciences and Psychology, Fairfield, USA; 3 Family Medicine, St. Bernards Medical Center, Jonesboro, USA; 4 Hematology/Oncology, St. Bernards Medical Center, Jonesboro, USA

**Keywords:** muscle pain, lung cancer, non small cell lung cancer, muscular metastasis, skeletal muscle mass

## Abstract

Non-small cell lung cancer metastasis to skeletal muscle is an uncommon occurrence. Lung cancers are more likely to spread to the brain, bone, liver, and adrenals. Here, we present a rare case of non-small cell lung cancer metastasis to the skeletal muscle in a 54-year-old male. In addition, we present a literature review on skeletal metastasis of non-small cell lung cancer. The most frequent presentation of skeletal muscle metastasis is muscular pain with or without swelling. The mechanism of metastasis to muscle is not well understood; it is theorized that hematogenous spread is the most likely route. As with our patient, the presence of skeletal muscle mass is considered an aggressive disease with poor survival, usually less than one year. The treatment for muscle metastasis is often palliative in the form of radiation therapy, chemotherapy, or surgical removal of the mass.

## Introduction

Although rare, non-small cell lung cancer can metastasize to skeletal muscle, which can present as muscle pain and swelling that at times may be correlated to a malignant process, especially in asymptomatic lung cancer patients. This phenomenon is thought to be uncommon due to the muscle’s innate protective mechanism against metastatic invasion through biomechanical damaging of tumor cells via anti-tumor factors [1].

A retrospective study done in 2014 revealed that muscle metastases (MM) from solid tumors include lung cancer at 25.1%, gastrointestinal tumors at 21.0%, and urological tumors at 13.2% [1], with evidence that radiological patterns and localization of MM vary in different primary tumors. Other studies also show that lung cancer is the most common malignancy with a hematogenous spread to the axial region of the body including the psoas muscle, gluteal muscles, and the paravertebral muscle. A painful palpable mass could be the only symptom prior to discovery of the primary cancer [2]; hence, it is crucial to differentiate between soft-tissue sarcoma and MM. If determined to be metastatic disease, further evaluation is needed to look for the primary.

## Case presentation

A 54-year-old male with a significant smoking history presented with slurred speech and left-sided upper and lower extremity weakness leading to a fall. Computed tomography (CT) of the head showed multifocal acute infarcts in the bilateral frontal, medial right parietal, and right cerebellar region suggesting an embolic etiology. Due to neck trauma from the fall, a CT spine was done that showed a right upper lobe mass with pleural invasion as well as mediastinal and hilar adenopathy. Embolic stroke with the incidental finding of lung mass suggested possible metastatic disease. On day 2 of admission, a CT of the chest/abdomen/pelvis showed possible metastatic disease in the right axillary node, bilateral adrenal gland nodules, and bilateral lytic lesions in the pelvis.

In addition, on day 2 of admission, the patient began complaining of right arm pain. As it was unclear whether this mass was associated with malignancy, an infectious process, or other etiology, a CT of the shoulder was obtained which revealed a heterogeneously enhancing mass in the lateral right deltoid without fluid collection or abscess. Given the patient's suspicion of metastatic disease, a biopsy of the right deltoid mass was obtained. Pathology revealed poorly differentiated carcinoma favoring non-small cell carcinoma, compatible with metastatic disease. Immunohistochemical staining showed strongly positive pancytokeratin and CK7 and negative for CD45, CK 20, TTF-1, and p40. Next-generation sequencing on the deltoid muscle mass reported PD-L1 80% and K-RAS G12C pathogenic variant. Subsequently, a bronchoscopy was performed with transbronchial needle aspiration of the right upper lobe tissue. Pathology of lung mass revealed non-small cell carcinoma with positive cytokeratin (AE1/AE3) and CK7.

**Figure 1:**
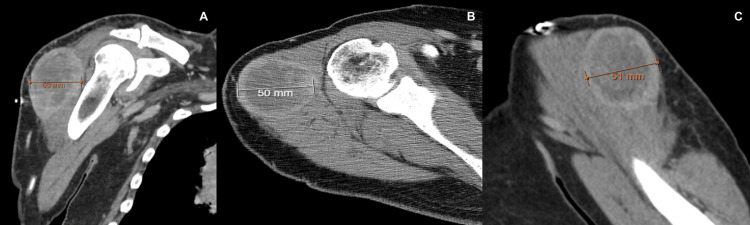
CT shoulder with contrast of R shoulder. (A) The sagittal view with the greatest dimension measuring 50 mm. (B) The axial view with the greatest dimension measuring 50 mm. (C) The coronal view with the greatest dimension measuring 51 mm.

Our index patient would have benefited from immunotherapy; however, given his poor performance status due to a recent embolic stroke likely related to metastatic disease with hemiparesis and aphasia, and his poor overall condition associated with a poor prognosis precluded him from being able to undergo aggressive treatment. Medical oncology recommended palliative or hospice care.

## Discussion

The purpose of this article is to emphasize the importance of evaluating arm/muscle pain in the setting of a concurrent incidental cancer diagnosis, as there is a chance it could be cancer-related and to review the literature for cases of skeletal muscle metastasis. In the case of our patient, the arm pain was overlooked until the patient had a stroke, which led to further workup and detection of cancer and subsequently identified as MM. Had this patient not had the stroke-like symptoms, the muscle pain may have been the only cancer-related symptom. This is a particularly important point in patients with risk factors for cancer without other symptoms and without prior guideline-directed cancer screenings.

Methodology

**Figure 2:**
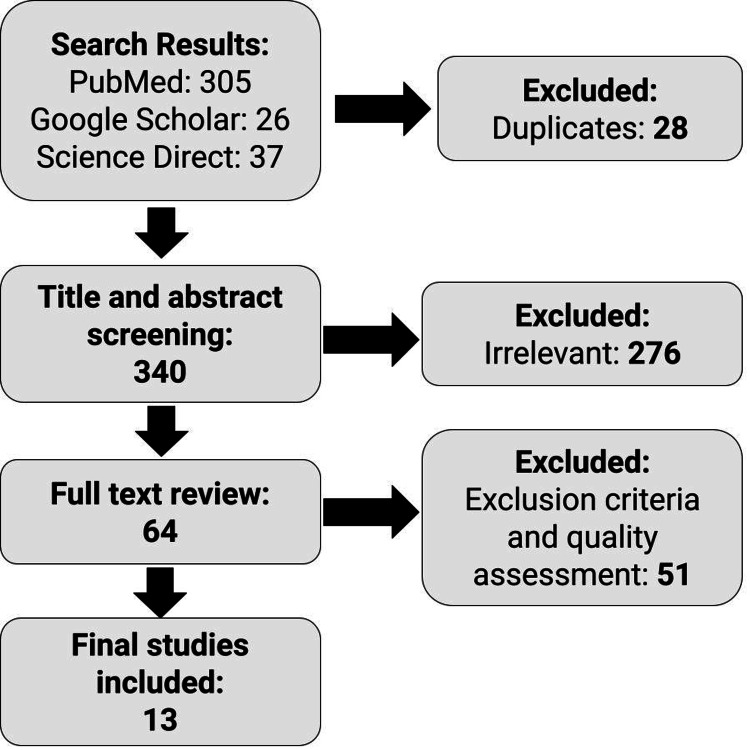
A flow chart showing a description of the studies selected for this literature review.

The Scale for the Assessment of Narrative Review Articles (SANRA) checklist was used to assess the quality of the studies included in this review article (Figure 2). Studies that scored 70% or more were included as final studies. The exclusion criteria included all studies in non-English languages, inaccurate data, and comorbidities that could bias the results.

Results

Table 1 summarizes the details of various studies included in this literature review.

**Table 1:** The results and conclusions of the studies included.

Author/Year	Type of Study	Findings/Conclusion
Acinas et al. [3]	Retrospective study	Fifty out of 190 neoplasms found on 723 autopsy results resulted in secondary involvement of skeletal muscle. Thirty-four cases had true macro/microscopic primary skeletal muscle metastases.
Sridhar et al. [4]	Case series	Hematogeneous metastasis to skeletal muscle is uncommon in non-small cell cancer and palliative radiation is the treatment of choice.
Sudo et al. [5]	Case report	Macroscopic metastases to muscle are infrequent, and four such cases were reported in a 15-year period.
Di Giorgio et al. [6]	Case series	Three cases of skeletal muscle metastases from lung cancer were reported with adeno-, squamous cell, and small cell carcinoma respectively with various anti-cancer regimens.
Razak et al. [7]	Case report with brief literature	Presentation of primary lung cancer in the form of skeletal muscle metastasis is unusual.
Pop et al. [8]	Retrospective data collection and literature review	Sixteen patients were identified in their department with skeletal muscle metastases, and the literature review suggested that this metastases is an aggressive disease, and metachronous metastatic deposits can be treated by surgery.
Kiara et al. [9]	Case report	A biopsy of the swollen muscle revealed the presence of pulmonary adenocarcinoma, making it an unusual presentation.
LeBan et al. [10]	Retrospective review	Muscle metastasis remains an atypical occurrence, because of its unique mechanical and metabolic qualities.
Agrawal et al. [11]	Case report	PET imaging showed metastasis to the right brachioradialis in a previously unknown primary right lung tumor with no pulmonary manifestations.
Sariaydin et al. [12]	Case report	Palliative radiotherapy could be a treatment option for skeletal muscle metastasis in stage 4 lung adenocarcinoma.
Syed et al. [13]	Case report with literature review	Skeletal muscle metastasis is an uncommon occurrence in non-small cell lung cancer, and treatment is mostly palliative with a poor prognosis overall despite a multimodal approach.
Peravali et al. [14]	Case report	Solid mass in the left gluteus muscle revealed poorly differentiated carcinoma, eventually leading to the diagnosis of non-small cell lung cancer.
Tuoheti et al. [15]	Retrospective review	A painful mass with an extensive peritumoral enhancement should be highly suspected to represent carcinoma metastasis to skeletal muscles.

Primary metastasis to muscle is still an uncommon phenomenon, considering muscle mass makes up roughly 50% of the body's entire weight. Although the incidence of skeletal muscle metastasis remains unknown, an autopsy series suggests that incidence may be closer to 0.08% [1, 2]. According to Tuoheti et al., out of 2,557 patients with lung cancer, only four individuals (0.16%) experienced skeletal muscle metastases [15]. Lung carcinoma seems to be the leading cause of skeletal muscle metastasis followed by gastrointestinal, prostate, bladder, kidney, pancreas, thyroid, breast, and ovary cancers [5].

Although the mechanism of metastasis to muscle is not well understood, it is theorized that hematogenous spread is the most likely route. However, despite accounting for half of the total body weight and having rich vasculature, muscular metastasis rarely occurs. It is possible that high resistance to muscle metastasis is due to contractility and muscle metabolism which leads to low pH, lactic acid buildup, and the presence of oxygen radicals creating an environment not conducive to tumor growth [3, 6].

The most frequent presentation of skeletal muscle metastasis is muscular pain with or without swelling, which is how our patient presented. For patients who continue to have muscle pain despite symptomatic treatment, clinical suspicion for other etiology including cancer should be explored, especially in patients with risk factors for developing malignancies and in patients who are not compliant with routine cancer screening.

Further diagnostic workup should include tetrahedron beam computed tomography, magnetic resonance imaging (MRI) scanning, ultrasound, and fine needle biopsy [5]. One literature review found that the most frequent findings on CT were homogeneous enhancement (type 1) [1]. In addition, Pretell-Mazzini et al. found that peritumoral edema was observed on MRI with carcinoma metastases. Thus, diagnosis can be problematic as the findings can be mistaken for an abscess or soft tissue tumors such as hemangioma, ganglion, or myxoma.

There is no established standardized treatment regimen or modification in therapy for the presence of skeletal muscle metastasis. Depending on the histologic type of the primary tumor and disease staging, chemotherapy and radiation therapy have been utilized in some cases. If drug-resistant pain from muscle metastases persists, surgical resection would be appropriate depending on the number, site, and dimensions, which are independent of the histologic type. Treatment for muscle metastasis is often palliative, and the presence of skeletal muscle metastasis is associated with poor survival, usually less than one year [10, 12, 13].

## Conclusions

The presence of MM indicates an aggressive illness. Even with radiologic imaging, diagnosing this condition can be challenging as findings are nonspecific and can be mistaken for soft tissue tumors or an abscess. Thus, the need for histopathologic diagnosis is paramount. By highlighting a differential diagnosis of cancer in patients presenting with an intramuscular tumor, this case contributes even more to the existing literature. Frequently, the outcome of treatment with chemotherapy, radiation, or even surgical resection is palliative. Thus, it is crucial to be vigilant in assessing treatment refractory muscle pain in patients with risk factors for cancer as it may be the only indication of an undiscovered malignancy.
